# The Impact of Storage Time and Reheating Method on the Quality of a Precooked Lamb-Based Dish

**DOI:** 10.3390/foods14152748

**Published:** 2025-08-06

**Authors:** Zhihao Yang, Chenlei Wang, Ye Jin, Wenjia Le, Liang Zhang, Lifei Wang, Bo Zhang, Yueying Guo, Min Zhang, Lin Su

**Affiliations:** 1 College of Food Science and Engineering, Inner Mongolia Agricultural University, Hohhot 010018, China; yangzhihao@emails.imau.edu.cn (Z.Y.); wcl2273859683@126.com (C.W.); jinyeyc@imau.edu.cn (Y.J.); 13154738132@163.com (W.L.); guoyy@imau.edu.cn (Y.G.); zmin@emails.imau.edu.cn (M.Z.); 2 Integrative Research Base of Beef and Lamb Processing Technology, Ministry of Agriculture and Rural Affairs of the people’s Republic of China, Hohhot 010018, China; 3 Xilin Gol League Animal Disease Prevention and Control Center, Xilinhot 026000, China; zhangl433523@163.com; 4 Xilin Gol League Qinmu Foods Co., Xilinhot 026000, China; wlf86123@163.com; 5 Inner Mongolia Daqi Foods Co., Ordos 017000, China; zhangbo3332025@163.com

**Keywords:** stewed lamb, refrigeration, freezing, microwaving, boiling, spoilage, cooking yield, water distribution, odor profile, flavor profile

## Abstract

Ready-to-eat meat products face quality challenges during storage and reheating. This study aimed to (i) characterize the physicochemical/microbiological changes in stewed mutton during storage (4 °C/−18 °C, 0–28 days) and (ii) evaluate reheating methods (boiling vs. microwaving) on day-7 samples. The nutritional analysis confirmed moisture reduction (57.32 vs. 72.12 g/100 g)-concentrated protein/fat levels. Storage at −18 °C suppressed microbial growth (the total plate count (TPC), 3.73 vs. 4.80 log CFU/g at 28 days; *p* < 0.05) and lipid oxidation (thiobarbituric acid reactive substances (TBARS): 0.14 vs. 0.19 mg/kg) more effectively than storage at 4 °C. The total volatile basic nitrogen (TVB-N) kinetics projected a shelf life ≥90 days (4 °C) and ≥120 days (−18 °C). Microwave reheating after frozen storage (−18 °C) maximized the yield (86.21% vs. 75.90% boiling; *p* < 0.05) and preserved volatile profiles closest to those in the fresh samples (gas chromatography–mass spectrometry (GC-MS)/electronic nose). The combination of freezing storage and subsequent microwave reheating has been demonstrated to be an effective method for preserving the quality of a precooked lamb dish, thereby ensuring its nutritional value.

## 1. Introduction

Lamb meat is highly appreciated by consumers due to its high nutritional value, as it is a source of protein and rich in indispensable amino acids, iron, and vitamin B12. In addition, mutton muscles may have advantages in terms of their tenderness and juiciness compared to other muscles [1]. *Eleutherine americana*, a bulb vegetable commonly called ‘red shallot’ in Asian cuisines, possesses a distinctive flavor profile and is nutritionally rich. It contains essential amino acids, trace elements (calcium, sodium, zinc, selenium, manganese), and bioactive compounds including niacin, allicin, and multiple vitamins. These constituents underpin its documented medicinal properties and health-promoting functions [2]. Therefore, *Eleutherine americana* is often cooked with lamb meat in China to make an *Eleutherine americana*-stewed lamb meat (EASM). In recent years, the demand for convenient, high-quality, ready-to-eat catering has grown rapidly with the increasing pace of life and changing consumer preferences. Ready-to-eat lamb-meat-based precooked dishes stand out for their unique flavor and nutritional value [3,4]. Making traditional EASM dishes into precooked dishes is an inevitable trend in adapting to today’s social structure and development, and it is of great practical significance to the development of the precooked dish industry. In order to guarantee that EASM precooked dishes meet the criteria for high nutritional, microbial, and sensory quality when delivered to consumers, it is crucial to investigate changes in the quality of these dishes during storage. Furthermore, the impact of reheating methods after storage must be examined. Refrigeration is widely used for quality preservation in foodstuffs [5] and is one of the most important strategies for maintaining the quality of food products during long-term storage [6]. Two main methods of refrigeration are used in the meat industry, chilling (0–5 °C) and freezing (−18 °C or less), effectively preventing the growth of most microorganisms and maintaining food quality [7]. However, refrigeration storage may alter the quality characteristics of meat, so we explored the effect of refrigeration temperature on the quality characteristics of EASM precooked dishes during storage.

In addition, EASM precooked dishes are mainly marketed as refrigerated precooked products that need to be reheated before consumption; boiling, steaming, and microwaving are a few common methods of reheating precooked meat dishes that are widely used in the commercial processing and food service industries [8]. Distinct heating principles generate physicochemical differences across reheating methods, measurable through an instrumental analysis of color parameters (L*, a*, b*), water-holding capacity (yield), and volatile flavor profiles (gas chromatography–mass spectrometry (GC-MS)/electronic nose), along with the organoleptic properties derived [9]. Luo et al. [3] found that microwave reheating exhibited better organoleptic properties and preserved the original flavor of surimi gelatin better compared to boiling, steaming, and frying reheating. Wang et al. [10] reported that a microwave-reheated Hongsu chicken precooked dish had the highest organoleptic scores and the least weight loss when compared to boiling it in hot water, steaming it, and oven-baking it. These results indicate that it is necessary to study the impact of reheating methods for EASM precooked dishes under different refrigeration conditions on their quality characteristics and flavor.

Therefore, this study pursued two distinct aims: first, to determine the impact of storage length (0–28 days) and refrigeration temperature (4 °C vs. −18 °C) on the chemical stability (lipid oxidation, moisture distribution) and microbiological quality (the total plate count (TPC) and total volatile basic nitrogen (TVB-N)) of EASM precooked dishes, and second, to evaluate the changes in their physicochemical properties (color, volatile profiles, yield) induced by the reheating method (microwave vs. boiling) in samples stored for 7 days under refrigeration (4 °C vs. −18 °C). In addition, the impact of two reheating methods on the color and flavor of the EASM precooked dishes under different refrigeration conditions was also evaluated. This study provides a theoretical basis for quality control of EASM precooked dishes.

## 2. Materials and Methods

### 2.1. Preparation of the EASM Precooked Dish

Fresh lamb leg meat was sourced from a local market (Dongwayao Market, Hohhot, China) and promptly placed in a culture box with ice packs for transport to the laboratory at 4 °C. Fresh Eleutherine americana was obtained from Inner Mongolia Daqi Food Co., Ltd. (Daqi Food Co., Ordos, China). The lamb meat, with a total weight of 5000 ± 5 g and cut into 2 cm cubes after removing external connective tissue and bone, was then boiled in water (5000 mL) with Eleutherine americana (325 g), ginger (150 g), and various seasonings (200 g). Following the removal of foam, the mixture was simmered in a pressure cooker (MX-ZZG01, Maoxin Machinery Co., Ltd., Weifang, China; 50 kPa) for 25 min. A total of 90 vacuum-sealed bags of the EASM precooked dishes were prepared. The cooked mixture was homogenized through stirring for 5 min and portioned into 100 ± 2 g units using a precision scale. Portions were vacuum-sealed (PA/PE bags, 90% vacuum rate) to yield 90 bags from a single batch, ensuring inter-bag consistency. These were divided into two batches: 60 bags for the storage experiments and 30 bags for the post-storage reheating experiments.

### 2.2. A Proximate Analysis of Raw Meat and Precooked Dishes

The moisture content was determined using the direct drying method, as outlined in “Determination of Moisture in Foods” (GB 5009.3—2016). The ash content was measured through muffle furnace incineration according to “Determination of Ash in Foods” (GB 5009.4—2016). The lipid content analysis employed the Soxhlet extraction method specified in “Determination of Fat in Foods” (GB 5009.6—2016). Protein quantification was performed using the Kjeldahl method described in “Determination of Protein in Foods” (GB 5009.5—2016). All analytical parameters were measured in triplicate.

### 2.3. Storage and Post-Storage Reheating

Storage experiments: The 60 bags of the EASM precooked dishes were randomly divided into two batches of 30 bags each. Each batch was stored for 0, 7, 14, 21, and 28 days, either under refrigerated (4 °C) or frozen (−18 °C) conditions.

Post-storage reheating experiments: The 30 bags of the EASM precooked dishes were randomly divided into five batches: (1) The control batch (C): Precooked samples without additional treatment. (2) The refrigerated–microwaved batch (RM): Samples were stored for 7 days at 4 °C and microwaved at 800 W (EG720KG3-NR1, Midea, Foshan, China) until their internal temperature reached 75 °C. (3) The refrigerated and boiled batch (RB): The samples were stored for 7 days at 4 °C and boiled until their internal temperature reached 75 °C. (4) The frozen and microwaved batch (FM): The samples were stored for 7 days at −18 °C and microwaved at 800 W until their internal temperature reached 75 °C. (5) The frozen and boiled batch (FB): The samples were stored for 7 days at −18 °C and boiled until their internal temperature reached 75 °C.

### 2.4. Storage Quality Analysis

#### 2.4.1. Microbiological Analysis

Ten grams of the EASM precooked dish sample from the storage experiment was added to 90 mL of sterile saline for homogenization. Continuous ten-fold dilutions of the mixture were then prepared using sterile saline and plated onto a plate count agar-based medium to determine the total plate count (TPC). The plates were incubated at 37 °C for 48 h. The number of colonies observed after incubation was recorded and expressed as log colony-forming units (CFU)/g [3].

#### 2.4.2. Spoilage Assessment

Total volatile basic nitrogen (TVB-N) was assessed using a slightly modified version of the method reported by Conway et al. [11]. First, 20 g of the sample from the storage experiment was minced and mixed with 100 mL of distilled water. After soaking for 30 min, the mixture was centrifuged at 3000 rpm for 10 min. The supernatant (1 mL) and saturated K_2_CO_3_ solution (1 mL) were then placed in the outer chamber of a Conway dish. Subsequently, 1 mL of boric acid solution (10 mM) and one drop of mixed indicator (1 mL of 1 g/L methyl red ethanol solution and 5 mL of bromocresol green ethanol solution) were added to the inner chamber of the dish. After incubating it at 37 °C for 120 min, the mixture in the inner chamber was titrated with 10 mM HCl until a pale pink color appeared. The TVB-N value (mg N/100 g) was calculated using the formula

TVB-N = [(V_1_ − V_2_) * C * 0.14 * d * 100]/m

where V_1_ = the titration volume of the test sample; V_2_ = the titration volume of the blank; m = the sample weight; C = the concentration of HCl; d = the dilution factor.

#### 2.4.3. Lipid Oxidation

The concentration of TBARS was measured using a slightly modified version of the method described by Tarladgis et al. [12]. In brief, 2 g of the EASM precooked dish sample from the storage experiment was minced and mixed with 3 mL of thiobarbituric acid (1%) and 17 mL of trichloroacetic acid-HCl (TCA-HCl, 2.5%). After adding butylated hydroxytoluene (BHT, 0.19 M, 0.5 mL), the mixture was heated in a boiling water bath (100 °C, HWS-26, Cany Precision Instruments Co., Ltd., Shanghai, China) for 30 min. After cooling to 25 °C, 4 mL of the resulting suspension was mixed with 4 mL of chloroform and vortexed for 1 min. The mixture was then centrifuged at 3000 rpm for 20 min. The absorbance of the supernatant was recorded at 532 nm. The TBARS value was expressed as follows:

TBARS (mg/kg) = A532 * 9.48/Ws

where A532 = the absorbance of the solution at 532 nm; Ws = the weight of the EASM precooked dish sample (g); and “9.48” is a constant derived from the dilution factor and the molar extinction coefficient of the red thiobarbituric acid reaction product (152,000 M^−1^ cm^−1^).

#### 2.4.4. Low-Field Nuclear Magnetic Resonance Application in this Study (Water Distribution)

Based on the method described by Bertram et al. [13], with slight modifications, the distribution of moisture in the EASM precooked dish from the storage experiment was analyzed using a low-field nuclear magnetic resonance (LF-NMR) analyzer (MesoMR23-040V-I, Niumai Analytical Instruments Co., Ltd., Shanghai, China). The samples (10 × 10 × 10 mm^3^) were wrapped in plastic film. The Carr–Purcell–Meiboom–Gill (CPMG) sequence was adopted to set the following transverse relaxation time (T2) parameters: the main frequency was set to 20 MHz, with an offset frequency of 796,572.84 Hz, a 90° pulse duration of 7 µs, and a 180° pulse duration of 13.52 µs. The sampling frequency was 100 kHz, with an RF delay of 0.08 ms, a digital gain of 3, and an analog gain of 20.0 dB. The data radius was set to 1, and 54,996 data points were sampled. The waiting time was 3000 ms, with 4 accumulations and an echo time of 0.24904 ms, resulting in a total of 49,800 signal sampling points.

#### 2.4.5. Shelf-Life Prediction

To predict the shelf life of the prepared vegetables, we applied two kinetic models to analyzing the TBARS and TVB-N data under isothermal conditions.

The first-order kinetic equation is expressed as

A = A0exp(kt)

where A is the quality indicator value at time t (days), A0 is the initial value at day 0, k represents the rate constant of change, and t is the storage time.

The Arrhenius equation is expressed as

k = k0exp(−Ea/RT)

Taking the logarithm of both sides,

lnk = lnk0 − Ea/RT

Here, k is the rate constant for the change in the quality indicator, k0 is the pre-exponential factor, Ea represents the activation energy (kJ/mol), T is the absolute temperature (K), and R is the gas constant (8.3144 J/mol·K). The parameters k0 and Ea are empirical constants related to the reaction system.

### 2.5. Reheating Quality Analysis

#### 2.5.1. Color Measurement

After reheating, surface color measurements were performed on three random locations of the cut muscle surface using a chroma meter (CR-400, Konica Minolta, Tokyo, Japan) in the CIE L* a* b* color space (Commission Internationale de l’Éclairage, 2004). The measurements used D65 illuminant, a 10° observer angle, and a 5.0 mm aperture, following ISO 11664-4:2019. The calibration of the device was conducted using a standardized white tile at the beginning of the analysis. Each sample in the reheating experiment was measured in triplicate.

#### 2.5.2. Cooking Yield

Yield was determined following Pathare et al. [14]. The initial weight (m1) of the EASM precooked dish from the reheating experiment was recorded before reheating. After reheating, the sample was allowed to cool at room temperature for 30 min. The surface moisture was blotted with a paper towel, and the weight (m2) was recorded. The yield of the EASM precooked dish was calculated as follows:

Yield (%) = m2 * 100/m1

#### 2.5.3. Chemical Analysis of the Odor Profile

The electronic nose analysis was mainly based on the method by Yu et al. [15]. The electronic nose analysis was conducted using the PEN3 system (Win muster Air sense Analytic Inc., Schwerin, Germany), which comprises 10 metal oxide semiconductors and sensors, as detailed in Table A1. Fascia was removed from the sample and minced. Then, 5 g of the sample from the reheating experiment was placed into a 15 mL vial and immersed in a constant-temperature water bath at 60 °C for 40 min, followed by a 1 h equilibration period at room temperature. During the electronic nose detection process, the parameters were set as follows: a sampling frequency of 1 Hz, an intake frequency of 400 mL/min, a cleaning time of 300 s, and a detection time of 120 s.

#### 2.5.4. Chemical Analysis of the Flavor Profile

The volatile flavor components of the samples were extracted using solid-phase microextraction (SPME), following the methodology outlined by Wettasinghe et al. [16]. Fascia was removed from the samples from the reheating experiment and minced into ground meat. The minced meat was then weighed to 5 g and transferred into 20 mL sample vials. Subsequently, the SPME probe (57310-U, Supelco Inc., Darmstadt, Germany) was inserted into the sample vial for adsorption under water bath conditions at 60 °C for 40 min. The needle was then removed and inserted into the injection port of the gas chromatography–mass spectrometry equipment (GC-MS; Thermo Fisher Scientific., Waltham, MA, USA). The dissolution process was carried out at 250 °C for 3 min. The following C conditions were used: a TR-5 column (30 m × 0.25 mm, 0.25 μm); high-purity helium was used as the carrier gas at a flow rate of 1.0 mL/min; and the temperature of the sample and the interface was maintained at 250 °C. A ramp-up procedure was used, starting at an initial temperature of 40 °C for 5 min, then ramping up to 200 °C at a rate of 5 °C/min for 5 min and then to 230 °C at a rate of 20 °C/min for 5 min. The conditions for MS included an electron ionization source with an ionization voltage of 70 eV, 250 °C for both the ion source and the transmission line, a mass scan range of 30–400 *m*/*z*, and a solvent delay of 1 min. For the qualitative and quantitative analyses, each peak in the GC-MS data of the total ion flux of known substances was searched for in the NIST (NIST14, version 2.2, National Institute of Standards and Technology, Gaithersburg, MD, USA) databases, and those with a match of more than 800 were qualitatively identified according to the laboratory database. The relative amount of each component was determined by normalizing the peak area.

### 2.6. The Statistical Analysis

All of the sample measurements were performed in triplicate, and the data are expressed as the mean ± standard deviation. Statistical analyses were carried out using IBM SPSS Statistics version 22.0 (SPSS Inc., Chicago, IL, USA) and R software version 4.3.2 (R Foundation for Statistical Computing, Vienna, Austria). The present study was based on two experiments: Experiment A: The impact of the storage conditions (two factors: storage time and storage temperature). A two-way ANOVA was applied to analyzing the data for this experiment. Experiment B: The impact of reheating (one factor: control and treatments for “storage temperature x reheating method”). A one-way ANOVA was conducted to analyze the data for this experiment. The first-order kinetic equation was analyzed and fitted using SPSS22.0 software, and the shelf life was predicted based on the Arrhenius equation. A principal component analysis (PCA) was performed on data obtained from the e-nose. Diagrams were generated using Origin 2023 (OriginLab Corporation, Northampton, MA, USA).

## 3. Results and Discussion

### 3.1. The Proximate Composition of the Raw and Cooked Meat

Table 1 compares the nutritional components of fresh lamb leg meat and the EASM precooked dish. The data indicate that the processed EASM dish exhibited a significantly reduced moisture content (57.32 vs. 72.12 g/100 g), alongside concentrated protein (32.35 vs. 21.26 g/100 g) and fat levels (7.37 vs. 3.81 g/100 g). These proportional increases reflect water loss during cooking rather than absolute nutrient gains—consistent with the effects of thermal concentration in cooked meats [17]. Minor modifications in the absolute protein/fat content may occur due to fat melting or soluble protein loss; however, moisture reduction is the factor that most significantly influences changes in composition. These results are consistent with the reported nutritional changes in heat-treated goat meat [17], demonstrating that the nutritional value of the EASM precooked preparation is effectively preserved.

### 3.2. The Effect of Storage on the Quality of the Precooked Dish

#### 3.2.1. The Effect of Storage Time on the Microbiological Characteristics of the EASM Precooked Dish

As shown in Table 2, the TPC of the ready-to-eat stewed lamb meat increased gradually during storage. This trend aligns with previous studies in beef, where microbial proliferation correlated with a decline in quality during extended storage [3,18]. The TPC of the EASM precooked dishes stored at −18 °C increased significantly within 0–7 days (*p <* 0.05), which was contrary to the research results of Luo et al. [3]. The reason for this result may be that the water content in the EASM precooked dishes became higher during 0–7 days, resulting in faster microbial reproduction. Notably, the TPC values for both batches were below the limit (≤5 Log CFU) according to the hygiene standard for cooked meat products (GB2627-2016). The TPC within 14–28 days of storage was significantly higher in the 4 °C batch than that in the −18 °C batch, which highlights the efficacy of freezing as a method for inhibiting microbial activity, thus slowing down the spoilage process in meat products [19]. Microbiologically, this temperature-dependent divergence aligns with known spoilage communities in refrigerated meats. Psychrotrophic genera like *Pseudomonas and Brochothrix*—common in lamb products—actively metabolize nutrients at 4 °C, while frozen storage (−18 °C) physically restricts microbial growth through ice matrix formation [19]. Though strain-level identification was not performed, the TPC kinetics suggest psychrotroph dominance in the refrigerated samples.

#### 3.2.2. Moisture Content, TVB-N, and Lipid Oxidation

**Figure 1:**
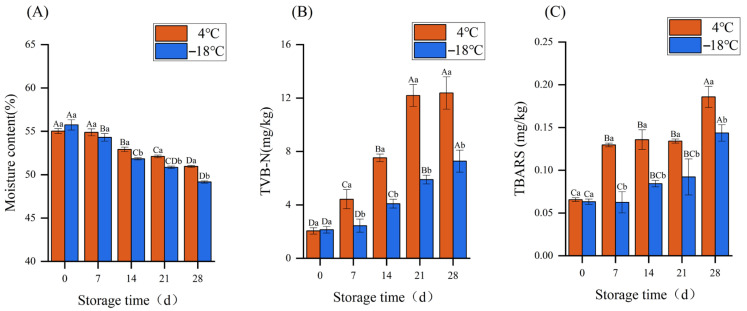
The effect of storage time and storage temperature on the quality of the EASM precooked dish. (**A**) Moisture content. (**B**) TVB-N content. (**C**) Lipid oxidation. Note: Lowercase letters indicate significant differences between the two temperatures (*p <* 0.05), and uppercase letters indicate significant differences between the number of days stored at the same temperature (*p <* 0.05).

A continuous decrease in moisture content was observed for all samples during the 28-day storage period (Figure 1). This reduction was more notable at −18 °C (11.84%) than at 4 °C (7.40%). A significantly higher moisture content was observed in the 4 °C batch compared to that in the −18 °C batch from day 14 (Figure 1A; *p <* 0.05). This disparity can likely be attributed to reduced mechanical damage in tissues stored at 4 °C, which helps preserve moisture. In contrast, ice crystal formation in frozen samples disrupts muscle fiber structure, impairing the meat’s water-holding capacity [20].

Compared to that at day 0, the TVB content was 6-fold higher in the refrigerated batch (4 °C) and 2-fold higher in the frozen batch (−18 °C). This is because lamb meat is a high-protein livestock product and is prone to quality changes such as microbial growth and spoilage during storage, which accelerates the degradation of nitrogen-containing compounds in meat products and leads to an increase in the TVB-N content [21,22,23]. Consistent with these findings, Wu et al. [24] demonstrated a significant increase in the TVB-N levels in tilapia mince over time. In this study, the TVB-N values were in line with the trends in microbial growth over the storage period, suggesting that increased microorganism numbers may contribute to a rising TVB-N content in EASM precooked dishes during storage. The TVB-N levels in the 4 °C batch were significantly higher compared to those in the −18 °C batch (*p <* 0.05). Consistent with our findings, Luo et al. [3] reported decreasing TVB-N levels in handled lamb meat as the storage temperatures decreased. This may be because low temperatures can slow down the growth and reproduction of microorganisms in the samples and reduce TVB-N production.

As shown in Figure 1C, the TBARS levels increased over the storage period in both batches, rising from 0.065 mg/kg on day 0 to 0.19 mg/kg on day 28 in the 4 °C batch and from 0.069 mg/kg to 0.14 mg/kg in the −18 °C batch. Critically, these remain well below the 1–2 mg/kg threshold, where rancidity renders meat organoleptically unacceptable. The refrigerated batch (4 °C) showed constant TBARS values from day 7 to day 21 of storage, which may have bene due to strong lipid oxidation in the EASM-prepared vegetables due to changes in temperature and a prolonged storage time [25,26,27]. The refrigerated batch (4 °C) exhibited significantly higher TBARS values compared to those in the frozen batch (−18 °C). Therefore, freezing slows down lipid oxidation in lamb meat. This conclusion was confirmed by other researchers in their studies [5].

#### 3.2.3. Water Distribution

**Figure 2:**
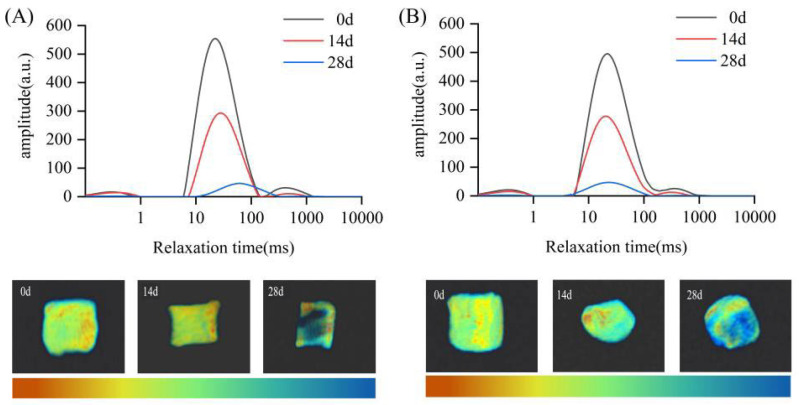
Changes in transverse relaxation times and pseudo-color images of the EASM precooked dishes according to storage time (0, 14, and 28 d) and storage temperature (4 °C (**A**) and −18 °C (**B**)). Note: The brighter the color in the image, the stronger the MRI signal.

Figure 2 displays the T2 relaxation time distributions of the precooked dishes under varying refrigerated storage times and temperatures. The T2 relaxation characteristics of the samples provide insights into water mobility, with the T2 value representing the water activity across different states. Specifically, T2 values are inversely correlated with the strength of water binding to macromolecules and directly correlated with the freedom of water movement. The three distinct T2 peaks correspond to three water states: tightly bound water (T21, 0–2 ms), less mobile water associated with the myofibrillar protein network (T22, 10–100 ms), and free water located in the interfibrillar spaces (T23, 100–1000 ms) [28,29].

The length of storage at −18 °C resulted in a marked decline in the T21, T22, and T23 peaks (Figure 2A). This indicates a reduction in the free water content, as well as the mobility of water and its availability within the frozen samples. This reduction is likely the result of ice crystal formation disrupting cellular structure and facilitating moisture loss. Additionally, the rightward shift in T22 and T23 suggests a gradual transition of less mobile water into free water, increasing its activity. This observation is consistent with previously reported results for frozen lamb meat samples [3]. On day 0, the samples exhibited extensive bright areas, which diminished by day 7, corresponding with a reduction in red coloration. By day 28, the majority of the sample appeared blue-green or dark, with only small regions remaining red or yellow, indicating a marked decrease in water retention under −18 °C storage. These changes corroborate previous studies [20] on the impact of freezing on water retention. Similarly, the T21, T22, and T23 peaks in the 4 °C batch declined over the storage period, although the overall leftward shift was less pronounced. This suggests that the state of water remained relatively stable, likely due to the absence of significant ice crystal formation under refrigeration, which preserved the integrity of the muscle fibers and minimized structural damage [30,31].

#### 3.2.4. Shelf-Life Modeling

According to Chinese regulations, meat products are considered unfit for consumption when their TBARS and TVB-N values exceed 2 mg/kg and 20 mg/kg, respectively. In this study, we utilized these critical quality indicators—TBARS, TVB-N, and TPC—to establish a shelf-life model for the EASM precooked dishes. The majority of the changes in food quality indicators during storage follow first-order kinetic equations [32]. The first-order kinetic equations for the TVB-N values in the EASM precooked dishes exhibited R^2^ values exceeding 0.921 across different temperatures (Table 3), demonstrating that these equations reliably modeled the TVB-N variations during storage.

Following this, the Arrhenius model was applied, as described by Wenjiao et al. [33], to refine the shelf-life predictions further. According to Table 3, the rate constants k for the TVB-N values were determined to be 0.0658 and 0.0488 at the different temperatures, with a regression slope for the TVB-N content of −960.72. The activation energy Ea was calculated to be 7.987 kJ/mol, while the pre-exponential factor k0 was found to be 0.745.

The TVB-N prediction model for the EASM precooked dishes is expressed as

A = A_0_*exp* [0.745 * (−7987/8.3144T)]t

where A is the predicted TVB-N content at a specific time, and A_0_ is the initial TVB-N content in the EASM precooked dish. This equation was refined further to develop a shelf-life prediction model based on the TVB-N content:

SL = [(lnA_1_ − lnA_0_)/0.745*exp*(−7987/8.3144T)]

where SL represents the predicted shelf life of the EASM precooked dish (days), A_1_ is the TVB-N threshold value at the end of the shelf life, and A_0_ is the initial TVB-N content in the EASM precooked dish. Using TVB-N kinetics, the provisional activation energy (Ea) was calculated as 7.987 kJ/mol. As a two-point estimate, this model assumes linear Arrhenius behavior without confidence intervals; non-Arrhenius effects (e.g., ice crystallization) may alter the kinetics. Using this equation, the conservative predicted shelf life determined based on the TVB-N content is at least 90 days at 4 °C and at least 120 days at −18 °C. Subsequent verification studies are needed to verify the reliability of the prediction model.

### 3.3. The Effect of Reheating Methods on the Quality Characteristics of the EASM Precooked Dish Under Different Storage Conditions

#### 3.3.1. Color and Yield

The results of this study showed that reheating conditions significantly influence the color attributes of the EASM precooked dish (Table 4). Each reheating method affected all of the color parameters, with a notable increase in the b* values post-reheating (*p <* 0.05). Specifically, the b* values for the FR batch (19.06) and the FM batch (18.54) were significantly higher than those for the other batches (*p <* 0.05), a change likely due to the exudation and oxidation of fat occurring during the reheating process [34]. In addition to the increase in b* values, the L* values, representing lightness, were significantly higher in the RB (60.21) and RM (56.51) batches compared to those in the C (53.52), FR (53.78), and FM batches (54.33) (*p <* 0.05). This increase in brightness can be attributed to the higher moisture content in the 4 °C batches, which released juices related to the low ability to retain water during reheating, brightening the surface of the lamb meat. The a* values, indicating redness, were also significantly influenced by the reheating method. The microwave-reheated batches (RM: 9.07; FM: 11.09) exhibited higher a* values within storage temperature than their water-bath-reheated counterparts (RB: 8.24; FR: 9.24) (*p <* 0.05), suggesting that the shorter reheating times associated with microwaving preserved the redness of the meat more effectively. Furthermore, reheating had a marked impact on yield. The yields for the microwave batches (RM: 86.53%; FM: 86.21%) were significantly higher than those for the BR batches (*p <* 0.05). This aligns with the findings of Wang et al. [10], who reported higher moisture retention in microwave-reheated meats. In contrast, the extended heating time in the BR batch led to increased water loss and the breakdown of muscle fibers. Additionally, fat cells ruptured, causing fat to flow into the broth. Simultaneously, the fat melted and decomposed during heating, further contributing to the reduction in yield [35,36].

#### 3.3.2. Odor Profile

**Figure 3:**
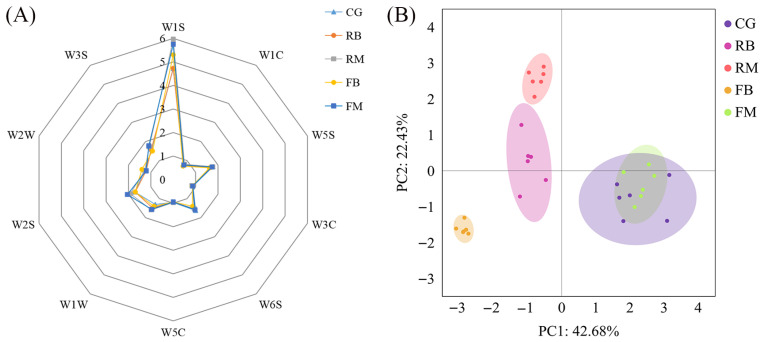
Electronic nose spider graph (**A**) and PCA scores (**B**) of the EASM precooked dishes according to the reheating method following refrigerated storage. C: control; RB: refrigerated and boiled; RM: refrigerated and microwaved; FB: frozen and boiled; and FM: frozen and microwaved.

As shown in Figure 3A, the odor response values under different reheating methods indicated that sensors W5S, W6S, W1S, W1W, W2S, W2W, and W3S all exhibited response values exceeding 1. This suggests that these sensors are particularly sensitive to the characteristic volatile compounds produced during reheating. Notably, the W1S sensor, which is sensitive to methyl compounds, demonstrated the highest response. The strongest odor responses were observed in the RM and FM batches, highlighting these reheating methods’ distinct impact on the volatile profile of the EASM precooked dish. To reduce the data complexity while retaining key information, a PCA was employed (Figure 3B). PCA helps visualize the similarities and differences among the samples based on their odor profiles. In this study, the first two principal components (PC1 and PC2) accounted for 46.68% and 22.43% of the total variance, respectively, leading to a cumulative contribution of 69.11%, thus capturing the majority of the information from the original dataset. Figure 3B shows the partial overlap between the FM and control C batches. This suggests that the microwave-reheated frozen samples retained flavor characteristics similar to those of the fresh samples. Among the tested methods, FM preserved the original odor profile of the EASM precooked dish best.

#### 3.3.3. Flavor Profile

Table 5 depicts the relative quantities of the volatile compounds identified in the samples subjected to reheating under various methods following storage under disparate conditions. A total of 30 compounds were identified, including 12 aldehydes, 9 alcohols, 4 ketones, 2 esters, and 3 other compounds. The majority of these compounds were attributable to lipid oxidation, Maillard reactions, thiamine degradation, and interactions between the resulting metabolites [37]. The signal intensities of each volatile compound are listed in Table 5. Due to their low odor threshold, aldehyde volatile flavor compounds significantly impact the aroma of cooked meat. In particular, branched-chain and small-molecule aldehydes, present in relatively high concentrations, contributed more to the flavor [38]. A total of 12 aldehydes were found in all of the sample batches, including pentanal, hexanal, benzaldehyde, octanal, (E)-2-Nonenal, and nonanal. Hexanal showed the highest peak intensity, with its levels in the RB, RM, FB, and FM batches being higher than those in the C batch (*p <* 0.05). This result aligns with Pratama et al.’s findings, which reported that hexanal is the most prominent aldehyde after meat is heated [39]. Compared to the other reheated samples, the RM samples exhibited significantly or numerically higher intensities for most aldehydes, possibly due to microwave processing accelerating lipid oxidation and producing more aldehydes, which contribute significantly to flavor [40]. These aldehydes are the primary aroma compounds in meat-related products. Specifically, hexanal imparts a grassy note and is mainly produced by the degradation of linoleic acid [41], octanal has a citrus-like odor and is formed by the oxidation of oleic acid [42], and pentanal has a cheesy smell [43]. Benzaldehyde, considered a linoleic acid degradation product, has fruity and woody aromas [44].

Ketones, which are primarily characterized by creamy or fruity aromas, are produced through the degradation of fatty acids as by-products of β-keto acid oxidation, enhancing the flavor profile of meat [45]. A total of four ketones were detected in all of the sample batches: 2-heptanone, 2,3-octanedione, 2-nonanone, and 2-undecanone. After reheating, the peak intensities of 2,3-octanedione, 2-nonanone, and 2-undecanone increased significantly (*p <* 0.05). Ketones are formed through the thermal oxidative degradation of unsaturated fatty acids or amino acids [46]. Their formation can be promoted by the thermal peroxidation of saturated fatty acids, keto-enol tautomerization of hydroperoxides, further oxidation of hydrocarbons, and decomposition or intramolecular electron rearrangement of peroxides from unsaturated fatty acids [46]. Changes in ketones after reheating can be presumed to affect the aroma characteristics of the EASM precooked dish. Additionally, the peak intensities of 2,3-octanedione and 2-undecanone in the RB and FB batches were significantly higher than those in the RM and FM batches (*p <* 0.05), likely due to prolonged water bath heating, which caused the oxidation of fats, impacting the flavor of the EASM precooked dish. This observation is consistent with the findings of Li et al. [47].

Similarly, alcohols are typically generated during the oxidative degradation of lipids, with unsaturated alcohols exerting a more significant influence on flavor attributes than saturated alcohols due to their lower odor thresholds [48]. A total of nine alcohols were detected across all sample batches, with 1-octen-3-ol, 2-ethyl-1-hexanol, and 1-octanol showing higher peak intensities. The peak intensities of 1-heptanol, 1-octen-3-ol, 2-ethyl-1-hexanol, 1-octanol, and isoborneol were significantly higher in the C batch than those in the reheated batches (RB, RM, FR, and FM) (*p <* 0.05), suggesting that reheating may have led to a reduction in the content of some if the volatile flavor compounds, thus affecting the flavor of the EASM precooked dish. Additionally, α-terpineol was detected in the EASM precooked dish, likely originating from the fennel, pepper, and cinnamon added [49]. The peak intensity of α-terpineol in the C, FR, and FM batches was significantly higher than that in the RB and RM batches (*p <* 0.05), possibly because storage and reheating at 4 °C led to further oxidation of α-terpineol, reducing its content. Esters in meat products mainly result from the esterification of carboxylic acids and alcohols [50]. Due to their relatively high odor thresholds, ester volatile flavor compounds contribute minimally to meat’s flavor [51]. Additionally, eucalyptol, endo-borneol, and camphor were identified as minor terpenoids. These compounds are usually derived from fragrance ingredients (e.g., bay leaf, star anise) [52]. Camphor, a terpene compound, is positively correlated with the addition of spices like garlic, black pepper, and white pepper, as reported by [53]. However, terpenes typically contribute little to meat’s flavor due to their high odor thresholds [54].

## 4. Conclusions

This work demonstrates that frozen storage (–18 °C) synergistically preserves the quality of ready-to-eat stewed lamb meat through dual mechanisms. Microwave reheating minimizes the quality degradation further by rapidly bypassing the protein denaturation thresholds, thereby retaining volatile compounds critical for sensory fidelity. These findings provide meat processors with a scientifically validated protocol—prioritizing frozen storage over refrigeration and microwave reheating over boiling—to extend products’ shelf lives while maintaining their organoleptic integrity. Future studies should scale this protocol to commercial production lines and evaluate the consumer acceptance across diverse demographics.

## Figures and Tables

**Table 1:** Proximate composition of raw lamb leg and precooked dish (g/100 g).

Parameter	Fresh Lamb Leg Meat	EASM Precooked Dish
Moisture	72.12 ± 3.23 ^A^	57.32 ± 1.72 ^B^
Ash	0.98 ± 0.21 ^A^	0.88 ± 0.17 ^A^
Protein	21.26 ± 0.7 ^B^	32.35 ± 0.32 ^A^
Fat	3.81 ± 0.35 ^B^	7.37 ± 0.45 ^A^

Note: Different letters in the same row indicate significant differences according to Student’s *t*-test.

**Table 2:** Changes in the microbiological quality of precooked dishes as a function of storage time and temperature. Data are expressed as Log (CFU/g).

Storage Time (d)	Storage Temperature	
	4 °C	−18 °C
0	<1	<1
7	2.72 ± 0.12 ^Ca^	2.68 ± 0.09 ^Ca^
14	3.91 ± 0.28 ^Ba^	2.98 ± 0.24 ^Bb^
21	4.32 ± 0.14 ^Aa^	3.53 ± 0.26 ^Ab^
28	4.8 ± 0.12 ^Aa^	3.73 ± 0.13 ^Ab^

Note: Lowercase letters indicate significant differences between the two temperatures (*p <* 0.05), and uppercase letters indicate significant differences between the number of days stored at the same temperature (*p <* 0.05). F = 12.34.

**Table 3:** Regression equations and parameters of TBARS, TVB-N, and TPC with time at different storage temperatures for the EASM precooked dish.

Testing Indicators	Absolute Storage Temperature/K	Regression Equation	Reaction Rate Constant K	Regression Coefficient R^2^
TBARS	277.15	y = 0.0549e^0.0353x^	0.0353	0.799
	255.15	y = 0.0852e^0.0229x^	0.0229	0.599
TVB-N	277.15	y = 2.525e^0.0658x^	0.0658	0.921
	255.15	y = 1.9589e^0.0488x^	0.0488	0.977

**Table 4:** The effect of reheating methods on the quality characteristics of the EASM precooked dish under different storage conditions (C (control), RB (refrigerated and boiled), RM (refrigerated and microwaved), FB (frozen and boiled), and FM (frozen and microwaved)).

Parameter	C	RB	RM	FB	FM
L*	53.52 ± 0.577 ^c^	60.21 ± 0.74 ^a^	56.51 ± 1.47 ^b^	53.78 ± 0.19 ^c^	54.33 ± 0.55 ^c^
a*	9.55 ± 0.11 ^b^	8.24 ± 0.37 ^c^	9.07 ± 0.48 ^b^	9.24 ± 0.11 ^b^	11.09 ± 0.67 ^a^
b*	13.48 ± 0.037 ^c^	17.72 ± 0.28 ^b^	17.66 ± 0.29 ^b^	19.06 ± 0.66 ^a^	18.54 ± 0.44 ^a^
Yield (%)	—	78.73 ± 2.31 ^b^	86.53 ± 3.15 ^a^	75.90 ± 1.97 ^b^	86.21 ± 2.36 ^a^

Note: Different letters in the same row indicate significant differences according to Duncan’s test (*p <* 0.05).

**Table 5:** The effect of reheating methods on the volatile flavor compounds in the EASM precooked dish under different storage conditions (C (control), RB (refrigerated and boiled), RM (refrigerated and microwaved), FB (frozen and boiled), and FM (frozen and microwaved)).

Compound	C	RB	RM	FB	FM
Aldehydes					
Pentanal	7.21 ± 2.64 ^c^	14.49 ± 0.25 ^b^	18.59 ± 0.80 ^a^	8.91 ± 0.20 ^c^	14.15 ± 2.59 ^b^
Hexanal	192.45 ± 75.59 ^b^	277.12 ± 12.56 ^a^	300.58 ± 11.63 ^a^	249.82 ± 30.85 ^a^	311.43 ± 51.52 ^a^
Benzaldehyde	17.40 ± 1.55 ^b^	26.03 ± 2.95 ^a^	29.45 ± 1.76 ^a^	26.41 ± 1.35 ^a^	19.01 ± 3.32 ^b^
Octanal	82.31 ± 11.74 ^a^	60.83 ± 2.10 ^c^	78.34 ± 2.23 ^b^	75.98 ± 0.80 ^b^	58.23 ± 3.19 ^c^
(E)-2-Nonenal	2.46 ± 0.55 ^a^	2.82 ± 0.33 ^a^	2.97 ± 0.07 ^a^	ND	2.35 ± 0.17 ^a^
Nonanal	128.96 ± 19.30 ^a^	94.44 ± 5.67 ^c^	125.56 ± 7.38 ^a^	111.11 ± 3.50 ^b^	99.06 ± 0.68 ^c^
Decanal	9.27 ± 1.34 ^a^	6.02 ± 0.35 ^b^	5.29 ± 0.74 ^b^	9.32 ± 1.33 ^a^	8.28 ± 0.34 ^a^
(Z)-2,6-Octadienal,3,7-dimethyl-	10.65 ± 2.55 ^bc^	9.13 ± 1.05 ^c^	8.55 ± 0.11 ^c^	17.47 ± 1.79 ^ab^	18.75 ± 5.61 ^a^
(E)-2,6-Octadienal,3,7-dimethyl-	12.04 ± 2.84 ^b^	27.16 ± 5.72 ^a^	10.79 ± 0.17 ^bc^	31.47 ± 2.16 ^a^	24.18 ± 5.87 ^a^
Citral	15.09 ± 0.39 ^d^	28.77 ± 1.63 ^c^	38.59 ± 0.97 ^b^	44.11 ± 1.32 ^a^	41.27 ± 0.34 ^a^
(E)-2-Decenal	ND	0.49 ± 0.13 ^b^	0.78 ± 0.03 ^a^	ND	ND
Hexadecanal	9.52 ± 1.32 ^b^	15.87 ± 2.73 ^a^	9.59 ± 1.65 ^b^	8.81 ± 1.56 ^b^	7.28 ± 1.93 ^b^
Ketones					
2-Heptanone	2.09 ± 0.45 ^a^	1.55 ± 0.01 ^b^	2.11 ± 0.01 ^a^	2.48 ± 0.16 ^a^	2.18 ± 0.24 ^a^
2,3-Octanedione	ND	125.53 ± 8.37 ^a^	110.98 ± 5.20 ^b^	133.69 ± 14.51 ^a^	96.25 ± 3.17 ^b^
2-Nonanone	3.11 ± 1.20 ^b^	5.91 ± 0.30 ^a^	5.23 ± 0.39 ^a^	5.14 ± 0.52 ^a^	5.37 ± 0.16 ^a^
2-Undecanone	1.85 ± 0.15 ^c^	5.58 ± 0.74 ^a^	2.70 ± 0.01 ^b^	5.83 ± 0.11 ^a^	3.11 ± 0.89 ^b^
Alcohols					
1-Heptanol	3.76 ± 1.29 ^a^	1.64 ± 0.22 ^b^	1.74 ± 0.01 ^b^	ND	ND
1-Octen-3-ol	68.59 ± 0.46	ND	ND	ND	ND
2-Ethyl-1-hexanol	277.38 ± 37.97 ^a^	56.46 ± 0.38 ^b^	53.84 ± 2.19 ^b^	54.92 ± 0.70 ^b^	23.86 ± 3.31 ^c^
α-Terpineol	11.73 ± 1.32 ^a^	4.72 ± 0.74 ^b^	6.89 ± 0.13 ^b^	12.89 ± 1.11 ^a^	13.78 ± 3.10 ^a^
2-Heptanol	ND	2.15 ± 0.05 ^a^	1.82 ± 0.08 ^b^	ND	ND
1-Octanol	91.98 ± 7.22 ^a^	29.23 ± 5.13 ^b^	23.60 ± 2.36 ^b^	11.14 ± 1.12 ^c^	9.69 ± 1.31 ^c^
Isoborneol	2.28 ± 0.14	ND	ND	ND	ND
2,4-Hexadien-1-ol	ND	ND	ND	2.65 ± 0.17 ^a^	2.74 ± 0.32 ^a^
2-Hexadecanol	0.23 ± 0.04 ^a^	0.20 ± 0.01 ^a^	0.26 ± 0.06 ^a^	0.20 ± 0.01 ^a^	0.19 ± 0.02 ^a^
Ethers					
n-Caproicacidvinylester	48.19 ± 5.65 ^a^	35.81 ± 5.57 ^b^	45.39 ± 6.58 ^a^	33.97 ± 9.02 ^b^	56.92 ± 12.54 ^a^
Decanoicacid,decylester	ND	ND	ND	5.52 ± 0.01 ^a^	3.09 ± 0.80 ^b^
Other					
Eucalyptol	46.08 ± 10.68 ^c^	87.80 ± 0.60 ^b^	79.87 ± 2.93 ^b^	106.69 ± 2.07 ^a^	96.49 ± 11.98 ^ab^
endo-Borneol	ND	23.96 ± 0.99 ^a^	ND	23.15 ± 1.32 ^a^	22.26 ± 4.19 ^a^
Camphor	ND	ND	ND	2.33 ± 0.17 ^a^	2.64 ± 0.59 ^a^

ND. Not detected. Note: Different letters in the same row indicate significant differences according to Duncan’s test (*p <* 0.05).

## Data Availability

The original contributions presented in this study are included in the article; further inquiries can be directed to the corresponding author.

## Appendix A

**Table A1:** Sensors used in the electronic nose (PEN3) and their performance.

Array Serial Number	Sensor Name	Performance Description
R1	W1C	Sensitive to aromatic components, benzene compounds
R2	W5S	Sensitive to nitrogen oxides
R3	W3C	Sensitive to aromatic components, ammonia compounds
R4	W6S	Sensitive to hydrides
R5	W5C	Sensitive to short-chain alkane aromatic components
R6	W1S	Sensitive to methyl compounds
R7	W1W	Sensitive to sulfides
R8	W2S	Sensitive to alcohols, aldehydes, and ketones
R9	W2W	Sensitive to organic sulfides
R10	W3S	Sensitive to long-chain alkanes
